# Supplementary figures and images for: Beneficial effects of cellular coinfection resolve inefficiency in influenza A virus transcription
Source: PLoS Pathog. 2022 Sep 19;18(9):e1010865. doi: 10.1371/journal.ppat.1010865 (PMC9521904; doi:10.1371/journal.ppat.1010865)

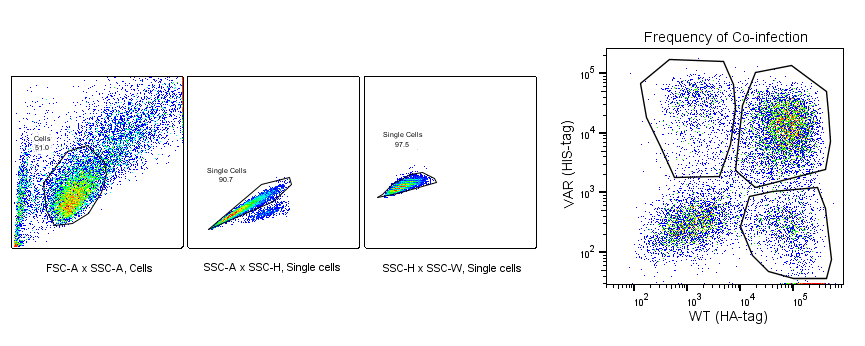

Supplement: S1 Fig — The ancestral gates for flow cytometry analysis of coinfection are shown, gating on cells, single cells, and, finally, sorting by epitope tag expression for uninfected, HA-tag+, HIS-tag+, and dual-tag+ cells. (TIF) [file ppat.1010865.s001.tif]

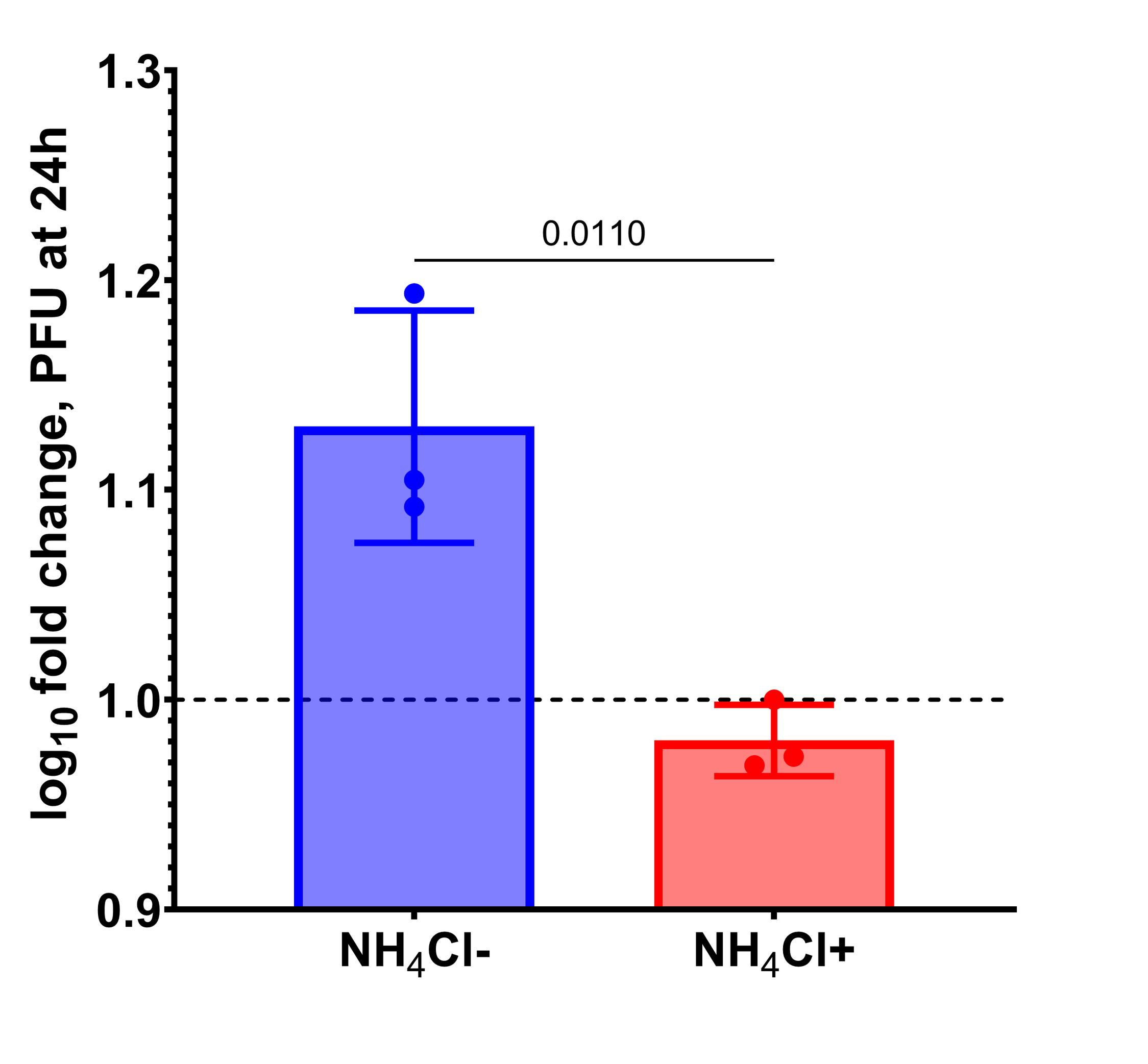

Supplement: S2 Fig — To confirm that the addition of 20 mM NH4Cl and 50 mM HEPES blocks infection, MDCK cells were inoculated with MaMN99 virus either in standard virus medium supplemented with trypsin or in virus medium lacking trypsin but including 20 mM NH4Cl and 50 mM HEPES. Viral replication was evaluated by titration of released virus sampled at 24 h post-inoculation. N = 3. Significance of differences was evaluated by unpaired, two-sided t-test. (TIF) [file ppat.1010865.s002.tif]
