# Supplementary material for: Beneficial effects of cellular coinfection resolve inefficiency in influenza A virus transcription
Source: PLoS Pathog. 2022 Sep 19;18(9):e1010865. doi: 10.1371/journal.ppat.1010865 (PMC9521904; doi:10.1371/journal.ppat.1010865)
Supplement: S1 Table — (DOCX) [file ppat.1010865.s003.docx]

**Supplementary Table 1:** Primers used for chimeric PA segment Gibson Assembly

| Oligonucleotide | Sequence |
| --- | --- |
| Endo GFHK99 F | ctgatccaaaatggaagactttgtgcgac |
| Endo GFHK99 R | attcgccccgggactgacgaaaggaatc |
| Endo pDP-MaMN99 F | tcgtcagtcccggggcgaatcaataattg |
| Endo pDP-MaMN99 R | agtcttccattttggatcagtacctgctttc |
| Arch GFHK99 F | ggatggattcgaaccgaacggctgcattg |
| Arch GFHK99 R | tctgaatccagcttgctagcgatctaggc |
| Arch pDP-MaMN99 F | gctagcaagctggattcagagtgaattc |
| Arch pDP-MaMN99 R | cgttcggttcgaatccatccacataggc |
| C-term GFHK99 F | gctagcaagctggatccagagtgagttc |
| C-term GFHK99 R | gccacaactattttagtgcatgtgtgag |
| C-term pDP-MaMN99 F | tgcactaaaatagttgtggcaatgctac |
| C-term pDP-MaMN99 R | tctggatccagcttgctagcgatctagg |
