# Supplementary material for: Beneficial effects of cellular coinfection resolve inefficiency in influenza A virus transcription
Source: PLoS Pathog. 2022 Sep 19;18(9):e1010865. doi: 10.1371/journal.ppat.1010865 (PMC9521904; doi:10.1371/journal.ppat.1010865)
Supplement: S2 Table — (DOCX) [file ppat.1010865.s004.docx]

**Supplementary Table 2:** Primers for ΔPA-X site-directed mutagenesis

| Oligonucleotide | Sequence |
| --- | --- |
| MaMN99PAX t627a F | gatttcaaatctttcttctattgtctcttcgcctctttcgga |
| MaMN99PAX t627a R | tccgaaagaggcgaagagacaatagaagaaagatttgaaatc |
| MaMN99PAX t597c_t600c F | ctctttcggactggcggaaggaatcccatagacccc |
| MaMN99PAX t597c_t600c R | ggggtctatgggattccttccgccagtccgaaagag |
| GFHK99PAX t627a F | atttcaaatctttcttctattgtctcttcgcctctctcgg |
| GFHK99PAX t627a R | ccgagagaggcgaagagacaatagaagaaagatttgaaat |
| GFHK99PAX t597c_t600c F | ctctctcggactggcggaaggaatcccatagacccc |
| GFHK99PAX t597c_t600c R | ggggtctatgggattccttccgccagtccgagagag |
