# Supplementary material for: Beneficial effects of cellular coinfection resolve inefficiency in influenza A virus transcription
Source: PLoS Pathog. 2022 Sep 19;18(9):e1010865. doi: 10.1371/journal.ppat.1010865 (PMC9521904; doi:10.1371/journal.ppat.1010865)
Supplement: S3 Table — (DOCX) [file ppat.1010865.s005.docx]

**Supplementary Table 3:** Primers used for strand-specific qPCR of MaMN99

| Oligonucleotide | Sequence |
| --- | --- |
| MaMN99 vRNA NS 552F | ggccgtcatggtggcgaat aatgcaattggaatcctcat |
| MaMN99 mRNA NS 13R | ccagatcgttcgagtcgttttttttttttttttatcattaaataag |
| MaMN99 NS 795F | cttgcaggcattgcaac |
| MaMN99 NS 643R | cggactccccaagcgaatctc |
