# Supplementary material for: Beneficial effects of cellular coinfection resolve inefficiency in influenza A virus transcription
Source: PLoS Pathog. 2022 Sep 19;18(9):e1010865. doi: 10.1371/journal.ppat.1010865 (PMC9521904; doi:10.1371/journal.ppat.1010865)
Supplement: Attachment — Submitted filename: Response to reviewers_Final.pdf [file ppat.1010865.s007.pdf]

Dear Drs. Brooke and Lopez,

We are pleased to resubmit a revised version of "Beneficial effects of cellular coinfection resolve inefficiency in influenza A virus transcription". We have made several changes to the text to address the reviewers' concerns and performed additional experimental analyses to determine genome copy:PFU ratios for our viruses and validate the use of NH_4Cl for blocking infection. The changes are outlined below. We have reproduced reviewers' comments and indicated our responses in blue font. We thank the reviewers for their thoughtful remarks and thank you for your time in handling our submission.

Sincerely,

Anice Lowen

Reviewer 1:

- Given the authors own expertise in working with avian cells, it would be of interest to measure the impact of this variant in avian cells.

    We agree that it would be of interest to examine how the PA K26E polymorphism affects viral phenotypes in avian cells. Contrary to the reviewer's speculation, we have not performed such experiments for the simple reason that we elected to focus our efforts on mammalian cells, where the extremely high reliance on multiple infection is seen for GFHK99. We are lacking some tools for avian cells (e.g. a minireplicon system) and choices had to be made to retain a reasonable scope for the project.

    We note, however, that existing published data on GFHK99 in avian systems offers relevant insight. We have expanded on our discussion of these points in the text, starting at lines 251-269 (and quoted below). We fully agree with the reviewer's proposed model that PA 26K has a greater impact in mammalian systems owing to a confluence of mal-adaptions.

    "Our previous work revealed that the extent to which GFHK99 virus relies on multiple infection for productive infection is strongly dependent on host species: in avian systems, the need for multiple infection was greatly diminished (1). Since we focused our studies herein on mammalian systems, our data do not reveal whether the GFHK99 PA enzyme is more active in avian cells or whether this avian virus is simply less sensitive to the cap-snatching deficiency in a host environment to which it is well-adapted. Nevertheless, prior work suggests the latter model is correct and that PA 26K is deleterious in both mammalian and avian hosts. First, PA 26K is proximal to the endonuclease active site and does not fall within interacting surfaces of defined cellular binding partners that might differ with host species. Second, PA 26K occurs rarely in sequenced strains, including those of the G1 lineage of H9N2 viruses (2) suggesting its occurrence in GFHK99 is the result of genetic drift. Third, introduction of PA K26E was seen to improve the kinetics and yield of GFHK99 viral replication in chicken cells and eggs (2). Fourth, GFHK99 variant viruses carrying PA K26E rapidly emerged and underwent positive selection in experimentally infected quail (3). Thus, while GFHK99 shows a reduced need for multiple infection in avian cells compared to mammalian cells (1), there is a fitness cost of PA 26K even in avian systems.

We therefore suggest that PA 26K has a greater impact in mammalian systems owing to a confluence of mal-adaptions to the mammalian cellular environment."

- Representative (or if possible all) flow cytometry data should be shown to show gating schema.

    Representative flow gating has been added as a Supplementary Figure 1.

- On an experimental note, I am a bit concerned by the way in which MOI was measured – if PA activity is attenuated, HA positivity above a threshold may no longer be a reliable read-out for infection. It would be good to see additional data confirming that for MOIs of GFHK99 and MaMN99 the authors are in fact comparing like with like.

    We agree that MOI measurement is an important consideration. Given that all IAV show some degree of reliance on multiple infection, any assay that works at limiting dilution will underestimate infectious potential. As the reviewer has identified, this issue is more extreme for GFHK99 than other viruses that we have examined, including MaMN99. For this reason, we have used genome copy numbers to define the multiplicity of infection in Figures 4 and 5, where viruses were directly compared at one or two discrete MOIs.

    In Figures 1, 2, 3 and 6, which evaluate the relationships between the frequency of HA+ cells and the frequency of dual-HA+ cells or the frequency of HA+ cells and reassortment, do not seek to compare across viruses at a given MOI but rather evaluate these relationships over a range of inoculation doses that span two orders of magnitude. Here, the need for higher MOI to achieve HA positivity (in the coinfection assay) or viral progeny production (in the reassortment assay) is exactly the effect we seek to document.

- I also liked the insistence on single cycle infections (line 83) and the method used to achieve this (line 384). However, as the authors note spill-over multi-cycle infections would distort the interpretation of the data, so it would be nice to see data showing that this method for limiting infections to a single cycle is robust

    To address this point we validated the method experimentally. The results are shown in Supplementary Figure 2 and outlined as follows at lines 388-396:

    "To verify the effectiveness of our method for limiting an infection to a single round of replication, we set up an experiment in which cells were infected with MaMN99 virus either within medium containing 20 mM NH_4Cl and 50 mM HEPES or with standard medium supplemented with TPCK trypsin (i.e. the medium used to allow 'multi-cycle conditions'). At 24 h post-infection, cell culture supernatant was sampled and infectious titers were determined by plaque assay. Results of this validation experiment showed that, in contrast to the multi-cycle conditions, no viral propagation was detected when single cycle conditions were imposed throughout the inoculation and growth stages of the experiment (Supplementary Figure 2)."

    Aside from this validation, we now include additional references in the text to indicate that NH_4Cl has been shown previously to prevent infection or reinfection:

- Neblock, D.S, and Berg, R.A. (1982) Lysosomotropic agents ammonium chloride and chloroquine inhibit both the synthesis and secretion of procollagen by freshly isolated embryonic chick tendon cells. Biochemical and Biophysical Research Communications 105(3): doi.org/10.1016/0006-291X(82)91055-5
- Prabhakara C, Godbole R, Sil P, Jahnavi S, Gulzar SeJ, et al. (2021) Strategies to target SARS-CoV-2 entry and infection using dual mechanisms of inhibition by acidification inhibitors. PLOS Pathogens 17(7): e1009706. https://doi.org/10.1371/journal.ppat.1009706

- Looking at the coinfection frequency, it looks non-saturated (ie the number of particles required to produce an HA+ event is less than the number at which coinfection measurements equal 100%). Given that it is non-saturated, my naïve understanding is that, assuming Poisson kinetics, you can place some kind of estimate on the average # of particles required to produce HA+. This might be a really nice number to share with the field. Not necessary, but would definitely be neat to have some kind of estimate.

  Although the assumption that Poisson statistics govern the distribution of virions into cells is reasonable, applying this to understand the observed HA positivity is not simple owing to the fact that coinfecting viruses interact such that HIS or HA tagged hemagglutinins are more likely to be detected when multiple viruses (of either type) are present. A fairly sophisticated model would need to be constructed that takes this interaction into account and jointly estimates parameters for i) the extent to which each virus augments the HA expression of another and ii) the number of viruses required to pass the limit of detection of HA expression. While this would be interesting to pursue, we feel it is outside the scope of the current manuscript.

- Figure 5 “high” and “low” MOI. The numbers are given in the methods, but why not just write what genome equivalents you are using here in the figure? If not in the figure body, maybe just in the legend?

  The specific values have been added to the legend to Figure 5: “Quantification of viral mRNA (F, H) and vRNA (G, I) in cells infected at low MOI of 0.5 RNA copies/cell (F,G) and high MOI of 1000 RNA copies/cell (H, I).”

- The authors, due to their genome-normalized infection schema, also have a genome/HA+ ratio. I definitely think the reliance on coinfection tells a compelling story, but I also think this ratio would be more immediately graspable in addition to the coinfection measurements. It would be nice if this number was presented to contextualize the coinfection data and show the skew of less infectivity per unit virus (with the coinfection data showing this is due to multiple-dose requirements).

  The reviewer makes a good point and we agree that most readers will relate well to the concept of genome/HA+ or similar ratio. Unfortunately, these data are not readily available since genome normalization and counting of HA+ cells were not used in the same experiments. Thus, to address the reviewer’s suggestion, we determined the genome/PFU ratio for a panel of our viruses, including MaMN99, MaMN99:GFHK99 PA and MaMN99:GFHK99 PA K26E. The data are now included as Table 1 and are discussed in the results section at lines 166-174.

Reviewer 2:

- I found the framing to be a little unexpected – mapping what appears to be a new (if currently rare) host determinant for influenza is interesting and rather underplayed, whereas the framing of the paper as a 'rare mechanistic insight' seems a bit overstated. The main finding (which is important on its own terms) seems to be that *any* mechanism that attenuates the virus would lead to an increased reliance on coinfection. This seems important conceptually rather than mechanistically (it could occur through many mechanisms, as indeed it does in this paper and as the authors note in line 307).

  The reviewer raises a good point and we agree that the larger concept is more important than the specific mechanism. Accordingly, we have reframed relevant sections of the abstract and discussion (and drafted an Author Summary that emphasizes the importance of MOI in defining fitness effects). Relevant text is quoted below.

  "**Abstract** …More broadly, the finding that deficiency in a core viral function is ameliorated through multiple infection suggests that the fitness effects of many viral mutations are likely to be modulated by multiplicity of infection, such that the shape of fitness landscapes varies with viral densities."

  "Author Summary
  Traditionally, a single virus particle is thought to be sufficient to initiate infection of a cell. However, the importance of coordinated infection, in which multiple viruses infect the same cell, has recently become apparent in diverse viral systems. With the goal of understanding how multiple infection increases the likelihood of productive infection, we investigated the traits that give rise to an extremely high reliance on multiple infection for influenza A/guinea fowl/HK/WF10/99 (H9N2) virus. We found that the phenotype maps to a single amino acid which results in a deficiency in transcription of viral mRNAs, a central function in the viral life cycle. This discovery suggests that the deleterious effects of many mutations that arise randomly within the influenza A virus genome may be alleviated when multiple viruses infect the same cell. In turn, coordinated infection is expected to be a major factor defining the evolutionary trajectories of viruses."

  "**Discussion [last paragraph]**… This work reveals that, in the specific case of GFHK99, multiple infection mitigates the deleterious effects of a specific mutation affecting a core viral process – transcription. It further suggests that multiple infection is likely to modulate the fitness effects of any mutation, such that the topology of fitness landscapes varies with viral population density. Finally, this effect is expected to be most potent under adverse conditions, such as in a new host species."

- As a mechanism of PA endonuclease attenuation the paper is really not very detailed, but as a concept the connection between attenuation and coinfection it is important – do the authors think that antiviral treatment might increase the risk of novel viruses emerging through reassortment, for example?

We thank the reviewer for raising this question. As noted in response to the comment above, we have extensively reframed the conclusions to emphasize the connection between attenuation and coinfection. Of course, any dynamic that favors coinfection will increase the probability of reassortment; that said, without significantly lengthening the Discussion, we do not see a natural place to address the specific example the reviewer gives here.

- While it was nice to use a modelled structure for the endonuclease domain of the virus in question, it would also be interesting to discuss this site in the context of the structures of the polymerase trimer during cap-snatching (albeit this would be the homologous site in a different viral strain). Does this give any more mechanistic explanation about what's going on here – or if not, does it identify a surface to which an as-yet-unidentified host factor might bind? (Or a known factor, such as the ANP32A binding site - do the authors currently have any thoughts on why this site might be host-type dependent?)

  We have examined the broader structure and sought connections of PA 26 to other identified interfaces between polymerase subunits, associated polymerases or host factors like ANP32A but have not to-date identified any that are likely to be impacted (see Extended data figure 7 in Phipps, et al. 2020). In our model of the folded protein, PA 26 is proximal to the PA active site, so perhaps there is a charge-dependent effect on this site.

  With regard to why polymorphism at PA 26 may have host dependent effects, our favored hypothesis is in line with that suggested by Reviewer 1 and now discussed in greater depth at lines 251-269. Briefly, we propose that PA 26K has a greater impact in mammalian systems owing to a confluence of mal-adaptions. (We direct the reviewer to the revised text quoted in response to Reviewer 1's first comment.)

- Line 10: 'additional viral templates' this implies that just adding more vRNA helps, which is an odd mental image – do the authors mean additional RNPs?

  Indeed, we used the term "template" to refer to the vRNP, since NP+RNA is the minimal template for the IAV polymerase. For clarity, we have added the word "ribonucleoprotein" in front of template in line 12.

- Line 113: please clarify how this was done to confirm that this mutation did not affect PA (if indeed this was the case)

  This method was designed and validated in Gaucherand et al. 2019. Two of the three mutations made to PA-X are nonsense mutations in the X-ORF that truncate PA-X and the third is a mutation to decrease ribosome slippage. None of these alter the coding potential of PA. Sequencing of the ΔPA-X reverse genetics plasmids confirmed that the PA coding sequence remained intact. This information has been clarified in the text at lines 361-364.

- Line 136: 'viruses displayed similar…' was there any statistical testing? What does similar mean here?

  We thank the reviewer for highlighting the need for statistical testing here. We performed non-linear regressions of the relationship between the percentage of cells infected and the percentage of cells coinfected. These regressions are now compared between the reference virus (GFHK99 PA or MaMN99 PA) and a test virus using the extra-sum-of-squares F comparison. This test essentially asks whether the same curve can be used to

model both sets of data. “Similar” in this case would mean that the two data sets are well represented by the same curve. For example, GFHK99 PA compared to MaMN99 finds the curves representing the two data sets to be significantly different ($p<0.0001$, $F= 47.59$) while the curve fit to GFHK99 PA compared with that of GFHK99 PA Endo is found to be the same ($p = 0.9385$, $F= 0.1356$). F test results are given in the legend to each figure where it has been applied.

- Fig 5G-I: relative to what?

  As indicated in the legend to Figure 5, the values plotted are relative to the viral mRNA or vRNA levels detected at 0 h post-infection.

- Line 243: ‘lowering the efficiency of viral transcription’ this is true, but there is a connected impact on host shut-off that could also be relevant

  We thank the reviewer for raising this point and fully agree. We added to Line 229: “Functionally, PA 26K reduces the activity of the endonuclease, lowering the efficiency of viral transcription and also impacting host shutoff capabilities of the virus”

- Line 248-9: ‘a positive density dependence’ – a bit more discussion would help here. To what extent is there evidence for (more limited) density dependence with WT viruses? What does it mean that the effect of adding more genomes seems greater when the virus is attenuated? Is it that following infection there is a progression towards a point when the cell is saturated, and that if the kinetics of that initial replication are slower there is more scope for additional genomes to make a difference? I appreciate that this might be speculative without further experimental work, but this is the main model that the paper develops and it would be useful to explore its implications in more depth here.

  We thank the reviewer for these ideas. We have expanded on ‘positive density dependence’ as an ecological principle and added the following text that seeks to address the reviewers’ questions (Lines 288-296):

  “We propose that the positive relationship between initial viral input and ultimate viral output arises because essential steps of the early viral life cycle are less prone to stochastic failure when the infecting population is larger. In unfavorable contexts - such as a new host species, antiviral treatment or deleterious mutation - the frequency with which these essential steps fail may be increased. In these situations, viral density would be expected to have a greater impact on viral growth rates. The acute density dependence of GFHK99 in mammalian systems is well explained by this paradigm and we expect these concepts apply broadly to IAV and many other viruses.”

- Line 280: The caveats about BXA are probably necessary, but a bit lengthy – I don’t think it’s particularly surprising that viral replication can proceed in the presence of an antiviral drug, and indeed all antiviral drugs tend to have a defined IC_{50}.

  The reviewer raises an excellent point about IC_{50}. We have removed some sentences from BXA explanation in the discussion and adjusted its location in manuscript (Lines 270-278).

- Line 343: ‘minimal’ – define (and briefly outline what the procedures were)

We have added more detail to this section at Lines 334-337: “Briefly, primers specific to terminal and internal regions of each segment were used in digital droplet PCR. Stocks were deemed low in defective interfering segments if the terminal:internal ratio was below a threshold of 4.”

- Line 348-9: the tag location should be explained more explicitly in a figure or table

  The tags have been explained more fully within the methods, including with the addition of a reference where the approach is described in more detail. Line 341: “The N-terminus of the HA segment of each virus used was engineered to contain either a 6xHIS or an HA epitope tag connected via flexible GGGS linker following the signal peptide. This approach was described previously and allows the signal to be present on mature HA proteins without interfering with the folding [30].”

- Line 384: what is the pH (this is critical)?

  We erroneously referred to the pH as high and have corrected this. The empirically determined pH of virus medium supplemented with 20 mM NH_4Cl and 50 mM HEPES is 7.10. This is noted at line 383.

  NH_4Cl supplementation has been used in other published research to prevent the infection of cells, in our case the re-infection of the cells with progeny viruses, by preventing acidification of endosomes.

- Line 239: ‘basis for these effects are’ -> ‘basis for these effects is’

  We have corrected this error.

- Line 249: ‘per capita’ – this tends to be used for counting people rather than viruses

  We removed “per capita”.
